# Di-μ-benzoato-κ^3^ *O*,*O*′:*O*;κ^3^ *O*:*O*,*O*′-bis­[aqua­(nitrato-κ*O*)(1,10-phenanthroline-κ^2^ *N*,*N*′)lead(II)]

**DOI:** 10.1107/S1600536812012974

**Published:** 2012-03-31

**Authors:** Yuanzheng Cheng, Fang Yan, Weiwei Shi, Liping Zhang

**Affiliations:** a Department of Chemistry, Weifang Medical University, Weifang 261053, People’s Republic of China

## Abstract

The title compound, [Pb_2_(C_7_H_5_O_2_)_2_(NO_3_)_2_(C_12_H_8_N_2_)_2_(H_2_O)_2_], crystallizes as a dinuclear centrosymmetric dimer containing two Pb^II^ atoms bridged by two benzoate ligands. Each Pb^II^ atom is seven-coordinated by a water mol­ecule, a nitrate anion, a 1,10-phenanthroline (phen) ligand and two benzoate anions. The crystal packing is stabilized by O—H⋯O hydrogen bonds and by π–π stacking between neighboring phen ligands, with a centroid–centroid distance of 3.557 (3) Å.

## Related literature

For related Pb(II) complexes with benzoate and 1,10-phenanthroline ligands, see: Dai *et al.* (2010 ▶); Li *et al.* (2011 ▶); Gao & Xuan (2009 ▶); Zhu (2006 ▶). 
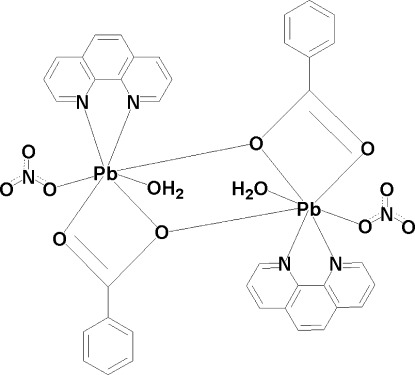


## Experimental

### 

#### Crystal data

[Pb_2_(C_7_H_5_O_2_)_2_(NO_3_)_2_(C_12_H_8_N_2_)_2_(H_2_O)_2_]
*M* *_r_* = 1177.08
Monoclinic, 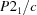

*a* = 11.7112 (13) Å
*b* = 13.5403 (15) Å
*c* = 14.1328 (11) Å
β = 124.483 (6)°
*V* = 1847.3 (3) Å^3^
*Z* = 2
Mo *K*α radiation
μ = 9.18 mm^−1^
*T* = 296 K
0.22 × 0.18 × 0.16 mm

#### Data collection

Bruker APEXII CCD area-detector diffractometer
Absorption correction: multi-scan (*SADABS*; Sheldrick, 1996) ▶ *T*_min_ = 0.150, *T*_max_ = 0.230
9109 measured reflections
3209 independent reflections
2788 reflections with *I* > 2σ(*I*)
*R*_int_ = 0.038

#### Refinement

*R*[*F* ^2^ > 2σ(*F* ^2^)] = 0.022
*wR*(*F* ^2^) = 0.051
*S* = 1.04
3209 reflections
262 parameters
6 restraints
H-atom parameters constrained
Δρ_max_ = 1.03 e Å^−3^
Δρ_min_ = −0.83 e Å^−3^

### 

Data collection: *APEX2* (Bruker, 2003 ▶); cell refinement: *SAINT* (Bruker, 2003 ▶); data reduction: *SAINT*; program(s) used to solve structure: *SHELXS97* (Sheldrick, 2008 ▶); program(s) used to refine structure: *SHELXL97* (Sheldrick, 2008 ▶); molecular graphics: *SHELXTL* (Sheldrick, 2008 ▶); software used to prepare material for publication: *publCIF* (Westrip, 2010 ▶).

## Supplementary Material

Crystal structure: contains datablock(s) I, global. DOI: 10.1107/S1600536812012974/lr2055sup1.cif

Structure factors: contains datablock(s) I. DOI: 10.1107/S1600536812012974/lr2055Isup2.hkl

Additional supplementary materials:  crystallographic information; 3D view; checkCIF report

## Figures and Tables

**Table 1:** Selected bond lengths (Å)

Pb1—O1	2.406 (3)
Pb1—O2	2.552 (3)
Pb1—N1	2.560 (3)
Pb1—N2	2.565 (3)
Pb1—O3	2.635 (3)
Pb1—O6	2.989 (3)
Pb1—O2^i^	2.913 (3)

Symmetry code: (i) 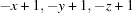.

**Table 2:** Hydrogen-bond geometry (Å, °)

*D*—H⋯*A*	*D*—H	H⋯*A*	*D*⋯*A*	*D*—H⋯*A*
O6—H6*A*⋯O4^ii^	0.85	2.29	3.116 (5)	164
O6—H6*B*⋯O3^i^	0.85	2.20	2.971 (7)	150

Symmetry codes: (i) 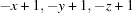; (ii) 
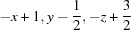
.

**Fig. 1.**
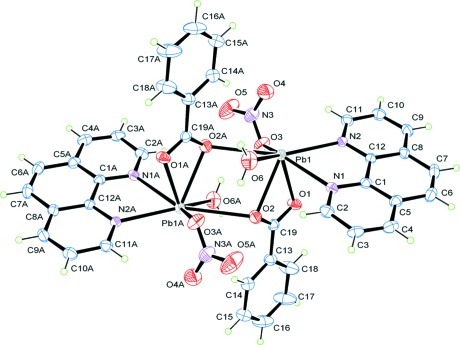
The molecular structure of the title compound. Displacement ellipsoids are drawn at the 30% probability level (symmetry code for atoms labelled A:-x+1,-y + 1, - z+1).

**Fig. 2.**
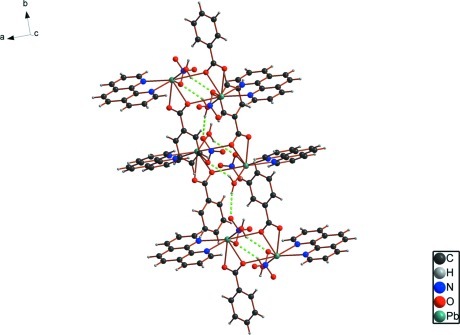
Part of the crystal structure of (I),showing O—H···O hydrogen bonds as green dashed lines.

**Fig. 3.**
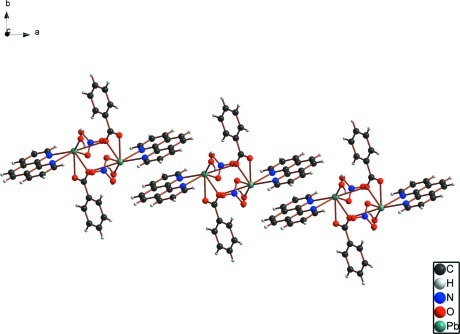
The π -π stacking of the 1,10- phenanthroline units in the title compound.

## supplementary crystallographic information

### Comment

Pb(II) complexes with benzoate and 1,10-phenanthroline ligands have been reported in the literature (Dai *et al.* 2010; Li *et al.* 2011; Gao & Xuan. 2009; Zhu 2006). Among the four complexes there is a coordination polymer (Zhu 2006), which contains nitrate ligand. In this paper, we report a new binuclear compound, which contains nitrate ligand too.

The crystal structure of the title complex consists of dimeric units [Pb_2_(NO_3_)_2_(C_7_H_5_O_2_)_2_(C_12_H_8_N_2_)_2_(H_2_O)_2_] (Figure 1),in which each Pb atom has a seven-coordinate geometry defined by one O atom donor from one water molecule,one O atom from a nitrate anion,two N atoms from 1,10-phenanthroline and three O atoms from two benzoate ligands. The Pb—N bond lengths and Pb—O bond lengths range between 2.560 (3) - 2.565 (3) Å and 2.406 (3) - 2.989 (3) Å (Table 1),The weak Pb—O bridging interactions form a four-membered Pb_2_O_2_ quadrilateral with Pb—Pb separation of 4.356 Å.

In the crystal, O—H···O hydrogen bonds link the water moleculars with nitrate ligands (Table 2 and Figure.2).Aromatic π-π stacking occurs between neighboring phen ligands (Figure.3).The centroid-centroid distance between C*g*1(N1/C1–C5) and C*g*2(N2/C8–C12) [symmetry code:-*x*,1 - *y*,1 - *z*] is 3.557 (3) Å.

### Experimental

A mixture of Pb(NO_3_)_2_(0.165 g,0.5 mmol), phenylsuccinic acid (0.097 g, 0.5 mmol), 1,10-phenanthroline(0.099 g, 0.5 mmol) and distilled water(15 ml) was sealed in a 25 ml Teflon-lined stainless autoclave. The mixture was heated at 423 K for 24 h. Then the autoclave was cooled to room temperature, after filtration, the resulting light red filtrate was allowed to stand at room temperature, and evaporation for 2 days afforded block light yellow single crystals. Phenylsuccinic acid was oxidized to benzoic acid under high temperature and pressure.

### Refinement

Aromatic H atoms were positioned geometrically and were included in the refinement in the riding model approximation, with C—H=0.93 Å and with *U*_iso_(H)=1.2*U*_eq_(C).Water H atoms were restrained at O—H=0.85 Å with *U*_iso_(H)=1.5*U*_eq_(O).

### Crystal data

**Table:**

[Pb_2_(C_7_H_5_O_2_)_2_(NO_3_)_2_(C_12_H_8_N_2_)_2_(H_2_O)_2_]	*F*(000) = 1120
*M**_r_* = 1177.08	*D*_x_ = 2.116 Mg m^−^^3^*D*_m_ = 2.116 (1) Mg m^−^^3^*D*_m_ measured by not measured
Monoclinic, *P*2_1_/*c*	Mo *K*α radiation, λ = 0.71073 Å
Hall symbol: -P 2ybc	Cell parameters from 6315 reflections
*a* = 11.7112 (13) Å	θ = 2.4–27.7°
*b* = 13.5403 (15) Å	µ = 9.18 mm^−^^1^
*c* = 14.1328 (11) Å	*T* = 296 K
β = 124.483 (6)°	Block, light-yellow
*V* = 1847.3 (3) Å^3^	0.22 × 0.18 × 0.16 mm
*Z* = 2	

### Data collection

**Table:**

Bruker APEXII CCD area-detector diffractometer	3209 independent reflections
Radiation source: fine-focus sealed tube	2788 reflections with *I* > 2σ(*I*)
Graphite monochromator	*R*_int_ = 0.038
phi and ω scans	θ_max_ = 25.0°, θ_min_ = 2.1°
Absorption correction: multi-scan (*SADABS*; Sheldrick, 1996)	*h* = −13→13
*T*_min_ = 0.150, *T*_max_ = 0.230	*k* = −15→16
9109 measured reflections	*l* = −16→16

### Refinement

**Table:**

Refinement on *F*^2^	Primary atom site location: structure-invariant direct methods
Least-squares matrix: full	Secondary atom site location: difference Fourier map
*R*[*F*^2^ > 2σ(*F*^2^)] = 0.022	Hydrogen site location: inferred from neighbouring sites
*wR*(*F*^2^) = 0.051	H-atom parameters constrained
*S* = 1.04	*w* = 1/[σ^2^(*F*_o_^2^) + (0.0209*P*)^2^ + 0.9758*P*] where *P* = (*F*_o_^2^ + 2*F*_c_^2^)/3
3209 reflections	(Δ/σ)_max_ = 0.003
262 parameters	Δρ_max_ = 1.03 e Å^−^^3^
6 restraints	Δρ_min_ = −0.83 e Å^−^^3^

### Special details

**Table:**

Geometry. All e.s.d.'s (except the e.s.d. in the dihedral angle between two l.s. planes) are estimated using the full covariance matrix. The cell e.s.d.'s are taken into account individually in the estimation of e.s.d.'s in distances, angles and torsion angles; correlations between e.s.d.'s in cell parameters are only used when they are defined by crystal symmetry. An approximate (isotropic) treatment of cell e.s.d.'s is used for estimating e.s.d.'s involving l.s. planes.
Refinement. Refinement of *F*^2^ against ALL reflections. The weighted *R*-factor *wR* and goodness of fit *S* are based on *F*^2^, conventional *R*-factors *R* are based on *F*, with *F* set to zero for negative *F*^2^. The threshold expression of *F*^2^ > σ(*F*^2^) is used only for calculating *R*-factors(gt) *etc*. and is not relevant to the choice of reflections for refinement. *R*-factors based on *F*^2^ are statistically about twice as large as those based on *F*, and *R*- factors based on ALL data will be even larger.

### Fractional atomic coordinates and isotropic or equivalent isotropic displacement parameters (Å^2^)

**Table:**

	*x*	*y*	*z*	*U*_iso_*/*U*_eq_	
Pb1	0.322274 (15)	0.530376 (10)	0.507038 (13)	0.03399 (7)	
O1	0.2190 (3)	0.5567 (2)	0.3057 (3)	0.0426 (7)	
O2	0.3808 (3)	0.4428 (2)	0.3797 (3)	0.0431 (7)	
O3	0.3786 (4)	0.7099 (2)	0.4726 (3)	0.0574 (9)	
O4	0.4076 (4)	0.7422 (3)	0.6335 (3)	0.0750 (11)	
O5	0.5305 (5)	0.8164 (3)	0.5870 (4)	0.1065 (16)	
N1	0.1221 (3)	0.4070 (2)	0.4019 (3)	0.0336 (7)	
N2	0.0825 (3)	0.6004 (2)	0.4347 (3)	0.0302 (7)	
N3	0.4402 (4)	0.7581 (3)	0.5646 (4)	0.0557 (10)	
C1	−0.0097 (4)	0.4381 (3)	0.3570 (3)	0.0319 (9)	
C2	0.1409 (5)	0.3132 (3)	0.3863 (4)	0.0439 (11)	
H2	0.2308	0.2909	0.4181	0.053*	
C3	0.0314 (5)	0.2464 (3)	0.3242 (4)	0.0467 (11)	
H3	0.0485	0.1816	0.3137	0.056*	
C4	−0.0996 (5)	0.2771 (3)	0.2796 (4)	0.0465 (11)	
H4A	−0.1732	0.2334	0.2384	0.056*	
C5	−0.1242 (4)	0.3755 (3)	0.2956 (4)	0.0389 (10)	
C6	−0.2605 (4)	0.4123 (4)	0.2514 (4)	0.0468 (11)	
H6	−0.3361	0.3700	0.2113	0.056*	
C7	−0.2807 (4)	0.5071 (3)	0.2672 (4)	0.0446 (11)	
H7	−0.3700	0.5296	0.2372	0.054*	
C8	−0.1654 (4)	0.5744 (3)	0.3301 (3)	0.0345 (9)	
C9	−0.1820 (4)	0.6735 (3)	0.3479 (4)	0.0400 (10)	
H9	−0.2698	0.6988	0.3185	0.048*	
C10	−0.0679 (5)	0.7327 (3)	0.4088 (4)	0.0434 (10)	
H10	−0.0771	0.7985	0.4221	0.052*	
C11	0.0622 (4)	0.6932 (3)	0.4507 (4)	0.0385 (10)	
H11	0.1389	0.7343	0.4923	0.046*	
C12	−0.0301 (4)	0.5400 (3)	0.3746 (3)	0.0295 (8)	
C13	0.2744 (5)	0.4860 (3)	0.1831 (4)	0.0407 (10)	
C14	0.3537 (5)	0.4185 (4)	0.1693 (4)	0.0511 (11)	
H14	0.4155	0.3773	0.2297	0.061*	
C15	0.3405 (6)	0.4129 (4)	0.0664 (5)	0.0624 (13)	
H15	0.3952	0.3690	0.0579	0.075*	
C16	0.2473 (9)	0.4715 (4)	−0.0234 (6)	0.080 (2)	
H16	0.2387	0.4668	−0.0928	0.096*	
C17	0.1666 (8)	0.5369 (4)	−0.0129 (5)	0.087 (2)	
H17	0.1037	0.5770	−0.0742	0.105*	
C18	0.1803 (7)	0.5426 (4)	0.0915 (5)	0.0721 (17)	
H18	0.1241	0.5859	0.0990	0.087*	
C19	0.2933 (4)	0.4950 (3)	0.2965 (4)	0.0333 (9)	
O6	0.4669 (4)	0.3437 (3)	0.6293 (3)	0.0666 (10)	
H6A	0.5130	0.3253	0.6990	0.100*	
H6B	0.4841	0.3147	0.5853	0.100*	

### Atomic displacement parameters (Å^2^)

**Table:**

	*U*^11^	*U*^22^	*U*^33^	*U*^12^	*U*^13^	*U*^23^
Pb1	0.03010 (10)	0.04089 (10)	0.03137 (11)	0.00026 (7)	0.01764 (8)	0.00064 (7)
O1	0.0506 (19)	0.0452 (16)	0.0353 (18)	0.0131 (14)	0.0263 (17)	0.0059 (13)
O2	0.0395 (17)	0.0530 (17)	0.0372 (18)	0.0071 (14)	0.0219 (16)	0.0072 (14)
O3	0.058 (2)	0.0539 (19)	0.041 (2)	−0.0079 (17)	0.0163 (18)	−0.0010 (17)
O4	0.073 (3)	0.092 (3)	0.059 (3)	0.008 (2)	0.037 (2)	0.001 (2)
O5	0.103 (3)	0.098 (3)	0.090 (4)	−0.064 (3)	0.038 (3)	−0.019 (3)
N1	0.0371 (18)	0.0358 (17)	0.0332 (19)	0.0023 (15)	0.0231 (17)	0.0000 (15)
N2	0.0287 (17)	0.0347 (17)	0.0257 (17)	0.0013 (14)	0.0144 (15)	0.0003 (14)
N3	0.056 (3)	0.051 (2)	0.047 (3)	−0.006 (2)	0.022 (2)	0.004 (2)
C1	0.037 (2)	0.036 (2)	0.026 (2)	−0.0030 (17)	0.020 (2)	0.0001 (16)
C2	0.051 (3)	0.037 (2)	0.049 (3)	0.003 (2)	0.032 (3)	−0.003 (2)
C3	0.061 (3)	0.033 (2)	0.042 (3)	−0.002 (2)	0.027 (3)	−0.0102 (19)
C4	0.056 (3)	0.042 (2)	0.038 (3)	−0.012 (2)	0.025 (3)	−0.008 (2)
C5	0.041 (2)	0.043 (2)	0.030 (2)	−0.0064 (19)	0.019 (2)	−0.0011 (18)
C6	0.035 (2)	0.059 (3)	0.038 (3)	−0.013 (2)	0.015 (2)	0.000 (2)
C7	0.029 (2)	0.058 (3)	0.046 (3)	−0.001 (2)	0.020 (2)	0.006 (2)
C8	0.031 (2)	0.046 (2)	0.030 (2)	−0.0001 (18)	0.019 (2)	0.0050 (19)
C9	0.036 (2)	0.048 (2)	0.041 (3)	0.011 (2)	0.025 (2)	0.007 (2)
C10	0.052 (3)	0.038 (2)	0.044 (3)	0.007 (2)	0.029 (2)	0.001 (2)
C11	0.043 (2)	0.036 (2)	0.042 (3)	−0.0034 (18)	0.027 (2)	−0.0052 (18)
C12	0.030 (2)	0.0354 (19)	0.026 (2)	−0.0014 (16)	0.0170 (19)	0.0008 (16)
C13	0.045 (3)	0.042 (2)	0.038 (3)	−0.003 (2)	0.026 (2)	−0.0020 (19)
C14	0.046 (3)	0.057 (3)	0.048 (3)	0.000 (2)	0.025 (3)	−0.008 (2)
C15	0.065 (3)	0.075 (3)	0.057 (3)	0.000 (3)	0.041 (3)	−0.014 (3)
C16	0.125 (6)	0.081 (4)	0.051 (4)	−0.016 (4)	0.060 (4)	−0.013 (3)
C17	0.139 (7)	0.075 (4)	0.045 (4)	0.038 (4)	0.051 (5)	0.013 (3)
C18	0.101 (5)	0.069 (3)	0.047 (3)	0.042 (3)	0.043 (4)	0.016 (3)
C19	0.032 (2)	0.0352 (19)	0.033 (2)	−0.0062 (18)	0.018 (2)	−0.0020 (18)
O6	0.059 (2)	0.069 (2)	0.071 (3)	0.0027 (18)	0.036 (2)	0.0086 (19)

### Geometric parameters (Å, º)

**Table:**

Pb1—O1	2.406 (3)	C6—C7	1.347 (6)
Pb1—O2	2.552 (3)	C6—H6	0.9300
Pb1—N1	2.560 (3)	C7—C8	1.443 (6)
Pb1—N2	2.565 (3)	C7—H7	0.9300
Pb1—O3	2.635 (3)	C8—C9	1.398 (6)
Pb1—O6	2.989 (3)	C8—C12	1.413 (5)
Pb1—O2^i^	2.913 (3)	C9—C10	1.366 (6)
Pb1—C19	2.837 (4)	C9—H9	0.9300
O1—C19	1.264 (5)	C10—C11	1.391 (6)
O2—C19	1.253 (5)	C10—H10	0.9300
O3—N3	1.255 (5)	C11—H11	0.9300
O4—N3	1.249 (5)	C13—C18	1.364 (7)
O5—N3	1.210 (5)	C13—C14	1.393 (6)
N1—C2	1.328 (5)	C13—C19	1.495 (6)
N1—C1	1.361 (5)	C14—C15	1.375 (7)
N2—C11	1.322 (5)	C14—H14	0.9300
N2—C12	1.364 (5)	C15—C16	1.366 (9)
C1—C5	1.398 (6)	C15—H15	0.9300
C1—C12	1.446 (5)	C16—C17	1.363 (9)
C2—C3	1.400 (6)	C16—H16	0.9300
C2—H2	0.9300	C17—C18	1.393 (7)
C3—C4	1.352 (6)	C17—H17	0.9300
C3—H3	0.9300	C18—H18	0.9300
C4—C5	1.407 (6)	O6—H6A	0.8500
C4—H4A	0.9300	O6—H6B	0.8501
C5—C6	1.435 (6)		
			
O1—Pb1—O2	52.46 (9)	C5—C6—H6	119.5
O1—Pb1—N1	74.22 (10)	C6—C7—C8	120.9 (4)
O2—Pb1—N1	79.07 (9)	C6—C7—H7	119.5
O1—Pb1—N2	76.72 (10)	C8—C7—H7	119.5
O2—Pb1—N2	124.16 (10)	C9—C8—C12	118.1 (4)
N1—Pb1—N2	64.77 (9)	C9—C8—C7	122.7 (4)
O1—Pb1—O3	69.88 (10)	C12—C8—C7	119.2 (4)
O2—Pb1—O3	95.94 (10)	C10—C9—C8	119.4 (4)
N1—Pb1—O3	137.55 (10)	C10—C9—H9	120.3
N2—Pb1—O3	85.27 (10)	C8—C9—H9	120.3
O1—Pb1—C19	26.28 (10)	C9—C10—C11	119.1 (4)
O2—Pb1—C19	26.20 (10)	C9—C10—H10	120.4
N1—Pb1—C19	75.79 (10)	C11—C10—H10	120.4
N2—Pb1—C19	100.98 (11)	N2—C11—C10	123.5 (4)
O3—Pb1—C19	81.72 (11)	N2—C11—H11	118.2
C19—O1—Pb1	96.3 (2)	C10—C11—H11	118.2
C19—O2—Pb1	89.7 (2)	N2—C12—C8	121.7 (3)
N3—O3—Pb1	110.3 (3)	N2—C12—C1	118.9 (3)
C2—N1—C1	118.0 (4)	C8—C12—C1	119.4 (3)
C2—N1—Pb1	122.5 (3)	C18—C13—C14	118.8 (4)
C1—N1—Pb1	119.5 (2)	C18—C13—C19	121.4 (4)
C11—N2—C12	118.1 (3)	C14—C13—C19	119.9 (4)
C11—N2—Pb1	123.1 (3)	C15—C14—C13	120.0 (5)
C12—N2—Pb1	118.8 (2)	C15—C14—H14	120.0
O5—N3—O4	121.1 (5)	C13—C14—H14	120.0
O5—N3—O3	121.1 (5)	C16—C15—C14	120.2 (5)
O4—N3—O3	117.9 (4)	C16—C15—H15	119.9
N1—C1—C5	122.5 (4)	C14—C15—H15	119.9
N1—C1—C12	118.0 (3)	C15—C16—C17	121.0 (5)
C5—C1—C12	119.5 (4)	C15—C16—H16	119.5
N1—C2—C3	122.8 (4)	C17—C16—H16	119.5
N1—C2—H2	118.6	C16—C17—C18	118.7 (6)
C3—C2—H2	118.6	C16—C17—H17	120.6
C4—C3—C2	119.3 (4)	C18—C17—H17	120.6
C4—C3—H3	120.4	C13—C18—C17	121.3 (5)
C2—C3—H3	120.4	C13—C18—H18	119.3
C3—C4—C5	119.8 (4)	C17—C18—H18	119.3
C3—C4—H4A	120.1	O2—C19—O1	121.4 (4)
C5—C4—H4A	120.1	O2—C19—C13	120.4 (4)
C1—C5—C4	117.5 (4)	O1—C19—C13	118.2 (4)
C1—C5—C6	120.0 (4)	O2—C19—Pb1	64.1 (2)
C4—C5—C6	122.5 (4)	O1—C19—Pb1	57.4 (2)
C7—C6—C5	121.0 (4)	C13—C19—Pb1	174.8 (3)
C7—C6—H6	119.5	H6A—O6—H6B	117.0
			
O2—Pb1—O1—C19	−1.6 (2)	C6—C7—C8—C9	179.7 (4)
N1—Pb1—O1—C19	−89.8 (2)	C6—C7—C8—C12	0.5 (6)
N2—Pb1—O1—C19	−156.9 (3)	C12—C8—C9—C10	−1.1 (6)
O3—Pb1—O1—C19	113.3 (3)	C7—C8—C9—C10	179.6 (4)
O1—Pb1—O2—C19	1.6 (2)	C8—C9—C10—C11	0.7 (6)
N1—Pb1—O2—C19	80.0 (2)	C12—N2—C11—C10	−0.5 (6)
N2—Pb1—O2—C19	31.0 (3)	Pb1—N2—C11—C10	177.9 (3)
O3—Pb1—O2—C19	−57.4 (2)	C9—C10—C11—N2	0.2 (7)
O1—Pb1—O3—N3	174.1 (3)	C11—N2—C12—C8	0.0 (6)
O2—Pb1—O3—N3	−139.6 (3)	Pb1—N2—C12—C8	−178.5 (3)
N1—Pb1—O3—N3	140.1 (3)	C11—N2—C12—C1	−179.6 (3)
N2—Pb1—O3—N3	96.5 (3)	Pb1—N2—C12—C1	1.9 (4)
C19—Pb1—O3—N3	−161.6 (3)	C9—C8—C12—N2	0.8 (6)
O1—Pb1—N1—C2	97.8 (3)	C7—C8—C12—N2	−180.0 (4)
O2—Pb1—N1—C2	44.0 (3)	C9—C8—C12—C1	−179.6 (4)
N2—Pb1—N1—C2	−179.7 (3)	C7—C8—C12—C1	−0.4 (6)
O3—Pb1—N1—C2	130.9 (3)	N1—C1—C12—N2	0.2 (5)
C19—Pb1—N1—C2	70.7 (3)	C5—C1—C12—N2	−180.0 (4)
O1—Pb1—N1—C1	−80.3 (3)	N1—C1—C12—C8	−179.4 (4)
O2—Pb1—N1—C1	−134.1 (3)	C5—C1—C12—C8	0.4 (6)
N2—Pb1—N1—C1	2.2 (3)	C18—C13—C14—C15	2.8 (8)
O3—Pb1—N1—C1	−47.3 (3)	C19—C13—C14—C15	−177.4 (4)
C19—Pb1—N1—C1	−107.5 (3)	C13—C14—C15—C16	−1.7 (8)
O1—Pb1—N2—C11	−101.9 (3)	C14—C15—C16—C17	0.5 (10)
O2—Pb1—N2—C11	−125.5 (3)	C15—C16—C17—C18	−0.4 (11)
N1—Pb1—N2—C11	179.5 (3)	C14—C13—C18—C17	−2.7 (9)
O3—Pb1—N2—C11	−31.5 (3)	C19—C13—C18—C17	177.5 (5)
C19—Pb1—N2—C11	−112.1 (3)	C16—C17—C18—C13	1.5 (11)
O1—Pb1—N2—C12	76.5 (3)	Pb1—O2—C19—O1	−2.8 (4)
O2—Pb1—N2—C12	53.0 (3)	Pb1—O2—C19—C13	177.0 (3)
N1—Pb1—N2—C12	−2.1 (2)	Pb1—O1—C19—O2	3.0 (4)
O3—Pb1—N2—C12	147.0 (3)	Pb1—O1—C19—C13	−176.8 (3)
C19—Pb1—N2—C12	66.4 (3)	C18—C13—C19—O2	−179.6 (5)
Pb1—O3—N3—O5	143.7 (4)	C14—C13—C19—O2	0.6 (6)
Pb1—O3—N3—O4	−34.4 (5)	C18—C13—C19—O1	0.2 (7)
C2—N1—C1—C5	−0.2 (6)	C14—C13—C19—O1	−179.6 (4)
Pb1—N1—C1—C5	178.0 (3)	C18—C13—C19—Pb1	−31 (3)
C2—N1—C1—C12	179.6 (4)	C14—C13—C19—Pb1	149 (3)
Pb1—N1—C1—C12	−2.2 (5)	O1—Pb1—C19—O2	−177.2 (4)
C1—N1—C2—C3	1.3 (6)	N1—Pb1—C19—O2	−94.1 (2)
Pb1—N1—C2—C3	−176.8 (3)	N2—Pb1—C19—O2	−154.3 (2)
N1—C2—C3—C4	−1.4 (7)	O3—Pb1—C19—O2	122.2 (2)
C2—C3—C4—C5	0.3 (7)	O2—Pb1—C19—O1	177.2 (4)
N1—C1—C5—C4	−0.8 (6)	N1—Pb1—C19—O1	83.1 (2)
C12—C1—C5—C4	179.4 (4)	N2—Pb1—C19—O1	22.9 (3)
N1—C1—C5—C6	179.3 (4)	O3—Pb1—C19—O1	−60.6 (2)
C12—C1—C5—C6	−0.5 (6)	O1—Pb1—C19—C13	33 (3)
C3—C4—C5—C1	0.7 (6)	O2—Pb1—C19—C13	−150 (3)
C3—C4—C5—C6	−179.4 (4)	N1—Pb1—C19—C13	116 (3)
C1—C5—C6—C7	0.6 (7)	N2—Pb1—C19—C13	56 (3)
C4—C5—C6—C7	−179.3 (4)	O3—Pb1—C19—C13	−28 (3)
C5—C6—C7—C8	−0.6 (7)		

Symmetry code: (i) −*x*+1, −*y*+1, −*z*+1.

### Hydrogen-bond geometry (Å, º)

**Table:**

*D*—H···*A*	*D*—H	H···*A*	*D*···*A*	*D*—H···*A*
O6—H6*A*···O4^ii^	0.85	2.29	3.116 (5)	164
O6—H6*B*···O3^i^	0.85	2.20	2.971 (7)	150

Symmetry codes: (i) −*x*+1, −*y*+1, −*z*+1; (ii) −*x*+1, *y*−1/2, −*z*+3/2.
